# Restage Transurethral Resection in the Evaluation of T1 High-Grade Bladder Tumor

**DOI:** 10.7759/cureus.103701

**Published:** 2026-02-16

**Authors:** Shibbir Ahmed, Mahabub Hassan, Tanzila Binte Jafar, M Rejwane Mahmud, Syed Ehsan Mahmud, Sayeedur Rahman, Ove Ahmed, A S M Anwarul Kabir

**Affiliations:** 1 Urology, Z. H. Sikder Women's Medical College &amp; Hospital, Dhaka, BGD; 2 Urology, Barts Health NHS Trust, London, GBR; 3 Pathology, Dhaka Medical College Hospital, Dhaka, BGD; 4 Urology, Kettering General Hospital NHS Foundation Trust, Kettering, GBR; 5 Urology, Sylhet MAG Osmani Medical College and Hospital, Sylhet, BGD; 6 Urology, North East Medical College and Hospital, Sylhet, BGD; 7 Urology, Sylhet MAG Osmani Medical College & Hospital, Sylhet, BGD; 8 Urology, Uttara Adhunik Medical College Hospital, Dhaka, BGD

**Keywords:** bladder cancer, muscle-invasive disease (t2, non–muscle-invasive bladder cancer (nmibc), residual tumor, restage/second-look turbt, t1 high-grade bladder tumor, transurethral resection of bladder tumor (turbt), tumor upstaging, urothelial carcinoma

## Abstract

Background: A considerable proportion of patients with T1 high-grade non-muscle invasive bladder cancer (NMIBC) advance to muscle-invasive disease, and the disease has a high rate of recurrence. Numerous approaches have been proposed in the literature to mitigate the consequences of the initial transurethral resection of bladder tumors (TURBT). We aimed to evaluate the T1 high-grade bladder tumor by restaging transurethral resection.

Methods: This was a descriptive observational study conducted in the Department of Urology, Sylhet MAG Osmani Medical College Hospital, Sylhet, from August 2023 to March 2024. A total of 41 patients with T1 high-grade urinary bladder tumors with the presence of a muscle layer in histopathology specimens were included in this study. Their demographic details, prior cystoscopy results, and histopathology reports were documented. A restaging TURBT was performed 2-6 weeks after the initial TURBT to reassess the T1 high-grade bladder tumor, focusing on the detection of any residual pathology.

Results: The study included 41 patients, mostly male (92.7%), with a mean age of 64.6 ± 6.6 years. After restaging, 41.5% (17 patients) had no residual tumor, while 58.5% (24 patients) had residual tumor. Of those with residual tumor, 20 (48.8%) remained T1, and 4 (9.8%) were upstaged to T2. The majority had high-grade tumors (51.2%, 21 patients). Residual tumor was associated with a higher incidence of tumor size >3 cm (16.0% vs. 6.3%) and multiple tumors (16.0% vs. 0.0%).

Conclusion: Restaging transurethral resection of a T1 high-grade bladder tumor detects residual tumor and upstaging of disease, which remains crucial for staging purposes and subsequent therapeutic effect.

## Introduction

Bladder cancer (BC) is the second most common urologic malignancy [1-4]. It mainly affects older adults: about 90% of cases are diagnosed after age 55, and roughly 80% after age 60 [5]. Globally, bladder cancer is more than four times as frequent in men as in women, with incidence rates of about 9.6 per 100,000 men [6]. Based on the World Health Organization (WHO) 2016 categorization, approximately 90% of bladder cancers are histologically of urothelial origin, while 1-7% of tumors are represented by squamous cell carcinoma [7].

The most widely used staging method is the Union for International Cancer Control's 2017 TNM classification of bladder cancer [8]. Tumors that involve the mucosa are determined as stage Ta, and those that invade the lamina propria are classified as stage T1 [9]. About 70-75% of bladder tumors are non-muscle invasive, and 25-30% are muscle invasive (T2 or higher) at presentation. Seventy percent of the non-muscle invasive bladder tumors are confined to the bladder mucosa (Ta), 25% invade the lamina propria (T1), and 5% are carcinoma in situ [10]. In India, 47% of bladder tumors are non-muscle invasive, and 36% of bladder tumors are muscle invasive, and among the NMIBC, 68% are high grade at presentation [11].

Per the European Association of Urology (EAU) guidelines, TURBT remains the cornerstone procedure for diagnosing and treating bladder tumors [12]. TURBT is used to verify the diagnosis, assess how deeply the tumor invades, and remove all visible lesions [13]. Non-muscle-invasive bladder cancer is difficult to manage because the disease can still have a strong potential for aggressive behavior [14].

Restaging TURBT included deep resection of the scar and edge of the initial resection site [15]. A second resection is considered to be of diagnostic, therapeutic, prognostic, and predictive value [16]. A restage TURBT should be performed in patients with T1 high-grade tumors before initiating maintenance intravesical chemotherapy or immunotherapy, and the interval between initial and restage TURBT should be within 2-6 weeks [15]. After the complete resection of the tumor in the initial TURBT, the tumor changes histopathologically in 71% of cases, and 53% of cases were upstaged to T2 (muscle invasion) at the second TURBT within six weeks [17]. According to a recent meta-analysis, more than 15% of patients with T1 bladder tumors had residual tumors following the initial, visibly complete resection [18]. In clinical settings, a restage TURBT may be the sole way to determine the tumor's mass, whether a muscle-invasive tumor is still there, or whether a comprehensive T1 high-grade tumor exists. All of this information has impacted the overall treatment of the disease [19]. Restaging TURBT has substantial clinical value. It can remove residual tumors that may drive recurrence, reduce overall tumor burden, and potentially lower recurrence rates and delay progression. This study aims to demonstrate the role of restaging TURBT in detecting residual disease, confirming the completeness of the initial resection.

## Materials and methods

Study design

This study was designed as a descriptive observational study conducted over a period from August 2023 to March 2024. The research was carried out at the Department of Urology, Sylhet MAG Osmani Medical College Hospital in Sylhet. The study population consisted of patients with a status of post-TURBT who presented with histopathological reports confirming T1 high-grade tumors. A purposive sampling technique was applied to select participants who met the specific study requirements.

Selection Criteria and Sample Size

The selection of cases was governed by strict inclusion and exclusion criteria to ensure the validity of the study. The inclusion criteria comprised post-TURBT patients whose histopathology reports indicated a T1 high-grade bladder tumor with the confirmed presence of a muscle layer. Conversely, patients were excluded if their tumor involved the prostatic urethra or trigone. Additionally, cases were excluded if the tumor involved the ureteric orifice or caused hydroureteronephrosis, ensuring that the study focused specifically on the target pathology without complicating anatomical involvement.

The sample size was estimated based on previously reported prevalence rates of residual tumor after restage TURBT, aiming to ensure adequate representation of outcome patterns rather than hypothesis testing [20], applying Zα = 1.96 for a 5% level of significance and Zβ = 0.84 for 80% power. Using a pooled prevalence of residual tumor of 56% (Naselli et al., 2018) [18] and assuming a 20% difference (p0 = 76%), the computed sample size was 38.4; after adding a 10% nonresponse rate, the final target sample size became 42.7 (approximately 43). A purposive sampling technique was used to recruit eligible patients during the study period. 

Preparation and intervention

To ensure consistency, specific operational definitions were adopted for the study. The grading of tumors was based on the degree of differentiation of tumor cells and the number of mitoses, which serve as presumed correlates of neoplasm aggressiveness. Staging was determined via clinical, radiological, and surgical criteria, including tumor size, regional lymph node involvement, and metastasis. Furthermore, a complete TURBT was defined as the resection of all visible tumors in their entirety, including resection down to normal-appearing muscle [21].

Some baseline investigation was done before the procedure, which included a urine routine examination, urine C/S, and USG of KUB with MCC & PVR. As anesthetic fitness was ensured in the initial TURBT, other investigations were rechecked by the anesthetist. After excluding any urinary tract infections, they were prepared for intervention. Operative findings of the initial TURBT (tumor size, number, and site) were documented in a preformed data collection sheet from the patient’s operation note. As per our institutional protocol, all patients received a single immediate postoperative intravesical instillation of mitomycin C within 24 hours of the initial TURBT, provided there was no suspicion of bladder perforation or significant postoperative bleeding. This was applied uniformly to the study population and was not stratified by age, tumor size, multifocality, or other clinical risk factors; these variables were recorded separately for analysis. Restage TURBT was done within 2-6 weeks of the initial TURBT, depending on the operating room availability.

Restage TURBT

Restage TURBT was performed by senior faculty urologists under general anesthesia with muscle relaxation in the lithotomy position after administration of a single prophylactic antibiotic dose. A 21 Fr cystoscope with a 30° telescope was introduced under white-light cystoscopy to identify the previous resection site (scar) and to inspect the entire bladder for any additional visible lesions. The cystoscope was then exchanged for a 26 Fr resectoscope. Resection/biopsy was performed using a standardized piecemeal technique with a loop electrode and continuous irrigation with 1.5% glycine (consistent with monopolar electrocautery use). The protocol at restaging focused on the prior tumor bed: a systematic resection/biopsy of the scar was undertaken with deep sampling of the base to include muscularis propria, followed by coagulation to secure hemostasis.

No enhanced optical imaging (e.g., NBI or HAL/blue light) and no laser energy were used. Irrigation was continued intraoperatively as needed to maintain adequate visualization and was discontinued at the end of the procedure once hemostasis was achieved. Postoperatively, a 22 Fr tri-channel Foley catheter was inserted, and continuous normal saline bladder irrigation was maintained until the effluent was clear, after which irrigation was stopped, and the catheter was removed per routine practice. Catheter traction was not routinely applied. Resected tissue was sent for histopathological examination, and findings were recorded.

Data analysis and ethical considerations

After data collection, all data were checked for completeness, consistency, and accuracy before analysis. Data were entered and analyzed using IBM Corp. Released 2020. IBM SPSS Statistics for Windows, Version 26. Armonk, NY: IBM Corp. Descriptive statistical methods were used for data analysis. Quantitative variables, such as age, were summarized using mean and standard deviation, along with minimum and maximum values. Qualitative variables, including sex, tumor stage, tumor grade, presence of residual tumor, tumor size, and number of tumors, were expressed as frequency and percentage. Cross-tabulation was performed to describe the distribution of residual tumor in relation to tumor size and the number of tumors at initial TURBT. No inferential statistical tests were applied, as the study was primarily descriptive in nature and intended to evaluate the distribution and pattern of findings following restage TURBT.

Prior to the commencement of the study, ethical approval was obtained from the Institutional Review Board (IRB) of Sylhet MAG Osmani Medical College. All participants were informed in detail about the objectives, methodology, potential benefits, and possible risks of the study in their local language. Written informed consent was obtained from each participant before enrollment. Participation in the study was entirely voluntary, and participants were informed of their right to withdraw from the study at any stage without affecting their treatment. Confidentiality of patient information was strictly maintained throughout the study. Data were anonymized and used solely for research purposes. No personal identifiers were disclosed, and all collected information was securely stored and accessed only by the investigator.

## Results

The study population comprised a total of 41 patients who had been diagnosed with T1 high-grade bladder tumors and subsequently underwent restage transurethral resection. An analysis of the demographic profile highlighted a marked male predominance within the cohort; specifically, there were 38 (92.7%) male participants compared to only 3 (7.3%) female participants. The age distribution of the subjects ranged from a minimum of 50 years to a maximum of 80 years. Upon categorization, it was observed that the largest proportion of patients fell within the 60-69 years age group, representing 21 (51.2%) individuals. This group was followed in frequency by patients aged 70 years and above, comprising 12 (29.3%) of the cases, while the 50-59 years age group accounted for the remaining 8 (19.5%) patients. Upon histopathological examination of the resected tissue, residual tumor burden was identified in 24 (58.5%) patients, while no evidence of residual malignancy was detected in the remaining 17 (41.5%) patients. Regarding the specific pathological staging outcomes observed after the second resection, 20 (48.8%) patients were confirmed to have persistent T1 stage disease. Of significant clinical importance, the restage resection was instrumental in detecting muscle-invasive disease in a specific subset of cases; consequently, 4 (9.8%) patients were formally upstaged to T2. Furthermore, in terms of tumor grading, high-grade pathology remained the predominant finding, identified in 21 (51.2%) patients, whereas low-grade pathology was observed significantly less frequently, found in only 3 (7.3%) patients (Table 1).

**Table 1:** . Patient characteristics and restage TURBT outcomes (n=41)

Domain	Category	n (%)
Age	50–59	8 (19.5%)
	60–69	21 (51.2%)
	≥70	12 (29.3%)
Gender	Male	38 (92.7%)
	Female	3 (7.3%)
Restage pathology (stage)	No residual tumor	17 (41.5%)
	T1	20 (48.8%)
	T2	4 (9.8%)
Residual tumor (restage TURBT)	No residual tumor	17 (41.5%)
	Residual tumor present	24 (58.5%)
Tumor grade (restage TURBT)	Low grade	3 (7.3%)
	High grade	21 (51.2%)

When analyzing the potential influence of tumor size, the data revealed distinct patterns between the groups. Among the patients who were found to have residual disease, the majority had presented with smaller lesions at the initial assessment; specifically, 21 (84.0%) of these cases involved initial tumors measuring less than 3 cm, while 4 (16.0%) patients had tumors larger than 3 cm. In contrast, within the cohort where no residual tumor was detected, larger lesions were notably scarce, with only 1 (6.3%) patient in this group having a tumor exceeding 3 cm.

The study further investigated the correlation between tumor multiplicity at the time of the initial surgery and the subsequent presence of residual disease. A detailed analysis revealed that among the patients in whom no residual tumor was found, 100% (n=16) had presented with a solitary tumor initially, with zero instances of multiple tumors recorded in this group. Conversely, a contrasting pattern was observed in the group with confirmed residual tumor, where 4 (16.0%) patients were documented as having multiple tumors at the initial TURBT. This distribution indicates that patients presenting with multiple tumors were exclusively confined to the residual disease category. The substantial overall detection rate of residual tumor in 58.5% of cases, coupled with the upstaging of 9.8% of patients, strongly underscores the clinical necessity of restage resection. These findings validate the critical utility of this procedure for achieving accurate disease stratification and ensuring the appropriate management of T1 high-grade bladder cancer (Table 2).

**Table 2:** Residual tumor at restage by initial TURBT tumor size and number

Initial TURBT factor	Category	No residual tumor	Residual tumor present
Tumor size	≤3 cm	15 (93.8%)	21 (84.0%)
	>3 cm	1 (6.3%)	4 (16.0%)
Tumor number	Single	16 (100.0%)	21 (84.0%)
	Multiple	0 (0.0%)	4 (16.0%)

## Discussion

The present study was conducted to evaluate the impact of restage TURBT for the T1 high-grade bladder tumor. The mean age of the patients was 64.6 ± 6.6 years, and most of the patients, 92.7% (n=38), were male. No residual tumor was found in 17 (41.5%) patients, and a residual tumor was found in 24 (58.5%) patients after re-stage TURBT. Whereas 48.8% of patients were in T1, and the rest, 9.8%, were in the T2 stage. Additionally, 51.2% of patients had high-grade tumors, while 7.3% of patients had low-grade tumors in the second stage of TURBT. A total of 4 patients had multiple tumors at initial TURBT, and 5 patients had tumors ≥ 3 cm. Among the patients without residual tumors, none of the patients (0.0%) had multiple tumors, whereas 1 (6.3%) patient had a tumor size >3 cm.

One of the characteristics of bladder cancer is that it primarily affects the elderly [22-27]. In this study, the mean age of the patients was 64.6 ± 6.6 years, and around half of the patients were in the 60-69 years age group. The retrospective study of Audenet et al. observed that the median age of the patients was 70 years [19]. Manoharan et al. reported the mean age of the sample was 56.4 years [28]. Eroglu et al. found it at 62.1 years [29]. 

BC affects over four times as many males as women. This gender disparity is most likely due to gender disparities in smoking tobacco [6]. Most of the patients in this study were male. Male predominance was observed in other studies [19,28-33].

Patients with high-grade non-muscle invasive bladder cancer (NMIBC) frequently have understaging at the time of the initial transurethral resection, which can cause a delay in a precise diagnosis and effective therapy [29]. About 23% of non-muscle-invasive bladder tumors eventually progress to muscle-invasive disease. Because understaging is possible, a repeat (restaging) TURBT is frequently advised in NMIBC to detect upstaging and guide any needed changes in treatment [34]. High-grade T1 non-muscle-invasive bladder cancer carries a substantial risk of disease progression, typically defined as progression to muscle-invasive disease (≥T2) and/or development of metastatic disease. In large series, the five-year progression risk for T1 high-grade disease is approximately ~20% (reported around 19.8%), and five-year disease-specific mortality is roughly ~10-12% (reported around 11.3%) [35].

After restaging TURBT, no residual tumor was found in 41.5% of patients, where 48.8% of patients were in T1, and the rest, 9.8%, of patients were in the T2 stage. In the prospective study of Shim et al., 75.9% had residual disease, whereas 10.3% of patients had ≥T2 after restaging TURBT of a T1 high-grade tumor [14]. The study by Manoharan et al. found 33.3% of patients had residual tumors on restaging [28]. The pooled prevalence of residual tumor was around 50.0%, while the overall upstaging to invasive disease was approximately 10% in the case of restage TURBT of a T1 high-grade tumor [18]. The presence of muscle in the initial TURBT reduces the remaining tumor rate after the second TUR. In research by Herr (1999), patients who did not have muscle in the initial TURBT had a larger risk of having a residual tumor at restage than those who had muscle layers (49% vs. 14%) [36]. In the current study, 51.2% of patients had high-grade tumors, while 7.3% of patients had low-grade tumors in the second stage of TURBT. In most cases, urothelial carcinomas show either low-grade or high-grade. However, in some cases, mixed patterns are also found. It was mentioned that any high-grade pattern, even a small focus, should be considered as high-grade. The findings of the present study were consistent with the histopathological report. These tumors should also be considered as high-grade.

Brausi et al. (2002) reported in their study that the rate of residual tumor increased from 7% of patients with a single tumor to 27-40% for patients with multiple tumors [37]. In the current study, multiple tumors were found in five patients. Among the patients without 24 residual tumors, none of the patients (0.0%) had multiple tumors, while among the patients with residual tumors, four (16.0%) patients had multiple tumors. Fan et al. reported in their study that 17.8% of patients with no residual tumor previously had a tumor ≥3 cm in diameter [38]. In this study, four tumors were more than 3 cm in diameter. Among the patients with no residual, only one (6.8%) patient had a tumor ≤3cm, and four (16.0%) patients had a tumor ≥3cm. In the study of Zurkirchen et al., the rate of residual tumor at second resection was 37% for beginners versus 26% for experienced surgeons [39]. Brausi et al. have also stressed the impact of the surgeon’s skills on the rate of residual tumor [37].

A restage TURBT has four goals: diagnostics, therapeutics, prognostics, and prediction. Indeed, studies have shown that a second TURBT can improve recurrence-free survival, give prognostic information, and predict outcomes following BCG treatment [19]. However, Calò et al. suggested that restage TURBT does not improve the oncological prognosis of patients with fully resected (muscle available and disease-free) high-grade T1 bladder tumors [40]. 

Herr shows in his study that the absence of muscle in the resected specimen was also found to be an important source of error. In its absence, the upstaging was 49%, and in its presence, it was only 14%. This finding underlines the importance of the assessment of muscle at first resection, which provides better staging. In the current study, in all patients who undergo restage TURBT, the muscle layer was present in the resected specimen of the initial TURBT [36].

This study has several limitations that should be considered while interpreting the findings. First, the study was conducted at a single tertiary care center with a relatively small sample size, which may limit the generalizability of the results to a broader population. Second, the absence of a comparative or control group restricts the ability to draw causal inferences or directly compare outcomes with patients who did not undergo restage TURBT. Third, the study employed a descriptive design without inferential statistical analysis, which limits the assessment of statistical associations between clinicopathological variables and study outcomes. Additionally, the interval between initial TURBT and restage TURBT varied between 2 and 6 weeks due to operation room availability, which may have influenced the detection rate of residual tumors. Finally, long-term outcomes such as recurrence, progression, and survival were not evaluated due to the lack of extended follow-up.

## Conclusions

Restage TURBT plays a crucial role in the management of T1 high-grade non-muscle-invasive bladder cancer by improving staging accuracy and confirming the completeness of the initial resection. In this study, residual tumor was detected in more than half of patients (58.5%) despite an initial TURBT reported as complete with muscle present in the specimen, highlighting the persistent risk of residual disease in this high-risk subgroup. Importantly, restage TURBT identified clinically significant upstaging to muscle-invasive disease (T2) in 9.8% of cases, a finding that would substantially alter subsequent treatment planning and supports the procedure’s value for optimal risk stratification.

Patterns observed in relation to initial tumor characteristics suggest that residual disease may be more frequent among patients with multifocal tumors and larger tumor sizes, reinforcing the need for careful evaluation of these features when planning further management. Overall, the high rate of residual tumor and the notable proportion of upstaging underscore that restage TURBT should remain a key component of standard care for patients with T1 high-grade bladder cancer, even when the initial resection includes muscularis propria. 
